# The children may not be the problem: evidence of acceptance and enjoyment of higher fibre breads from choice architecture studies in school breakfast clubs

**DOI:** 10.1098/rstb.2024.0151

**Published:** 2025-09-18

**Authors:** Nicholas Wilkinson, Eloise Tann, Neil Boyle, Samantha Caton, Victoria McColl, Fiona Croden, Gurpinder Singh Lalli, Louise Dye

**Affiliations:** ^1^ School of Food Science and Nutrition, University of Leeds, Leeds, UK; ^2^ School of Psychology, The University of Sheffield, Sheffield, UK; ^3^ Sheffield Centre for Health And Related Research, Division of Population Health, School of Medicine and Population Health, The University of Sheffield, Sheffield, UK; ^4^ AB Mauri UK and Ireland, London W1K 4QY, UK; ^5^ School of Psychology, University of Leeds, Leeds, UK; ^6^ Faculty of Education, Health, and Wellbeing, University of Wolverhampton, Wolverhampton, UK; ^7^ Institute for Sustainable Food, The University of Sheffield, Sheffield, UK

**Keywords:** children, diet, fibre, school breakfast, system change

## Abstract

Low fibre consumption is endemic in the United Kingdom (UK). Replacing refined staples with wholegrain starchy staples could increase fibre consumption. School food contributes to children’s nutrition and establishes eating norms, presenting both a public health opportunity and challenge. It could be a policy lever to effect generational dietary change and influence health outcomes, and goal-strategic public sector procurement. However, this policy lever is under-exploited. Despite efforts by non-governmental organizations and individual schools, school food has not been conferred high value or status in national food policy and often does not provide children with a diet rich in healthy, high-fibre foods. In the H3 project, we trialled interventions to increase the fibre content of school breakfast, with particular focus on replacing white bread—a UK breakfast staple—with higher fibre breads. Here we review this work, providing insights from the food industry and children’s perspectives. A key outcome was that child preferences were not a major barrier. When provided with higher fibre foods, children ate and enjoyed them. This suggests that simple food policy levers could significantly reduce the approximately 6 g average ‘fibre gap’ in UK school children’s intake, for example by mandating that all bread served in schools be at least a ‘source of fibre’. Larger scale trials should be conducted, ideally as part of universal school breakfast pilots.

This article is part of the theme issue ‘Transforming terrestrial food systems for human and planetary health’.

## 1. The state of the nation: children in need, and the role of schools

The incumbent United Kingdom (UK) Labour government’s Child Health Action Plan states: ‘There is no more important indicator of the state of a country than the wellbeing of its children’ ([1, p. 1]). Across the UK, childhood levels of obesity and overweight continue to rise [2–5]. Overconsumption of energy-dense, micronutrient-poor foods can result in children living with both obesity and nutritional deficiencies, known as the ‘double burden of malnutrition’ [6]. Consumption of essential nutrients decreased or fell below levels recommended by Public Health England between 2008 and 2019 [7]. Children face the greatest risk of insecure access to nutritious food [8]. According to UK government figures, approximately 2.4 million children (17% of UK children) lived in food-insecure households in 2022−2023 [9]. Unequal access and availability of good, nutritious diets are reflected in obesity and type-2 diabetes prevalence rates that are higher in low-income populations [7,10,11].

Fibre is essential for human health and should form a part of all children’s diets [12]. Inadequate fibre intake in children is associated with constipation, irritable bowel syndrome and immune-related disorders [12,13]. Increasing evidence shows an inverse relationship between adult fibre intake and colorectal cancer, type II diabetes, cardiovascular disease (CVD), all-cause mortality and mortality from CVD, cancer and coronary heart disease [14–17]. There is also growing recognition of the role of fibre in metabolic health and obesity via the gut microbiome [11]. The percentage of children and adolescents meeting the UK Scientific Advisory Committee on Nutrition 2015 [18] recommended daily intake of fibre was low (<15%) [7], especially in low-income populations [19], during the measurement period 2008−2017. There is therefore a strong interest in strategies to increase fibre consumption [20].

Dietary behaviour in childhood sets the foundation for dietary behaviour in adulthood [21–24] and can be challenging to change during childhood [25–27]. When faced with competing foods and free choice, young children often opt for high-energy-dense and familiar alternatives [28–30]. One way to improve acceptance of bitter, sour or novel/disliked foods in children is to increase familiarity via repeated exposure, demonstrated to be a robust approach including for wholegrain foods [31,32]. Typically, eight to ten exposures are required before acceptance occurs [33]; however, carers will often decide that their child does not like a particular food after only five offerings [34]. Despite its robust efficacy, repeated exposure could falter because of food waste that occurs during the initial exposures. This is especially problematic for low-income families. Offering food in nurseries and schools avoids this problem and maximizes population impact, and capitalizes on observational learning, social modelling and social praise [35].

The healthy soil, healthy food, healthy people (H3) project is a multidisciplinary integrated programme of research at laboratory, farm, landscape, local, regional and national scales, funded by UKRI’s ‘Transforming UK Food Systems’ programme [36]. Here, we report on work within H3 to increase fibre consumption in school breakfast provision. We also present an industry perspective from the baking supplies company AB Mauri (see Industry perspective, boxes 1, 2 and 3). Beyond purely nutritional benefits, breakfast is associated with improved classroom behaviour, and cognitive and academic performance [38,39], though establishing causal relationships is difficult [40]. Equally, the size of the school food market could provide a powerful policy lever to encourage the development of healthy foods aimed at children (see box 1). Schools in England can access support through the Department of Education funded National School Breakfast Programme (NSBP) [41] and non-governmental organization programmes such as Magic Breakfast (https://www.magicbreakfast.com). The NSBP offer covers 75% of breakfast food and delivery costs, whereas Magic Breakfast requires a membership fee. Eligibility is based on the proportion of pupils from disadvantaged backgrounds. Some schools run their own breakfast clubs or employ external third parties. Whole-school approaches to food are recommended [42], but can in practice be difficult owing to the complex environments in which schools function [43]. Thus, we focus here on simple, scalable interventions that are relatively accessible to any school.

Box 1. **Industry perspective: investment in innovation for healthier diets is happening across the food industry, but commercially can be a risk.**

In 2023, the Food and Drink Federation reported that the food industry allocated an estimated £160–£190 million towards the development of healthier products, as detailed in their ‘Innovation for Healthier Diets’ report [37]. Within the packaged bread sector, substantial efforts to innovate new varieties that successfully balance high fibre content with appealing taste and texture have been expended. This is achieved through the use of diverse grains, seeds and other high-fibre ingredients. The consumer market is inundated with a plethora of options, from hybrid bread varieties to dense wholegrain loaves. However, the demand for higher fibre breads in this format remains relatively niche. This limited appeal has resulted in high-fibre products being withdrawn from the market shortly after their introduction.

Box 2. **Industry perspective: white bread sales dominate the ambient bread market.**

According to Kantar Worldpanel’s Traditional Ambient Bread reports for the 52 weeks ending on 17 March 2024, 19 March 2023 and 20 March 2022, white bread continues to dominate the traditional ambient bread segment, accounting for 66.6% of the total spend share with this trend showing an upward trajectory from 2022 through 2024.

Box 3. **Industry perspective: the challenge of making healthier innovations accessible to children.**

Numerous technological advancements and ingredient options are available that support the reformulation of bread to make it both higher in fibre and appealing to children. However, it is important to note that these enhanced products often come with a higher price tag. This makes them inaccessible for underfunded government or charity initiatives such as school breakfast or lunch programmes, where the primary goal is to feed children. In the competitive landscape of the bakery industry, manufacturers lack commercial incentives to provide alternatives to basic white bread for school food services. This is particularly the case when these entities are engaged in competitive pricing, which may hinder the widespread adoption of healthier alternatives.

Typically, UK breakfasts feature starchy cereal-based foods, with bread/toast and ready-to-eat breakfast cereals being most popular [44]. Breakfast consumption frequently consists of low-fibre starchy foods such as white bread, including in schools [45]. Replacing these with higher fibre alternatives offers a significant target [46–48]. White bread remains the most widely consumed bread [47,49]. Breakfast cereal consumers already benefit from relatively higher fibre consumption [50]. Therefore, we focused on bread while also considering other aspects of school breakfast provision, such as breakfast cereals and fresh fruit.

Following a similar programme introduced by the Welsh government in 2022 [51], in 2024 the UK Labour government confirmed its commitment to fund free 30 min, before school breakfast clubs in all primary schools in England, with a pilot scheme in over 700 schools beginning in April 2025 [52]. This offers an important opportunity to promote higher fibre provision when the scheme is fully implemented. Currently, school dietary regulations and guidelines encourage higher fibre options alongside low-fibre options [53]. This choice architecture implicitly passes the responsibility from the adult provider to the child; if children are not eating enough fibre, it is because they are choosing the ‘wrong’ foods, not because gatekeepers are supplying them with the ‘wrong’ foods—‘wrong’ in this sense meaning the food that provides the lowest amount of fibre. In this programme of research, we explored the acceptability of higher fibre foods and the feasibility of choice architectures that offer only higher fibre choices. We suggest this approach is fairer for the children and may be more effective as a mode of increasing fibre consumption.

## 2. The dominant myth: ‘the children will only eat white bread’

Industry is responding to calls for higher fibre breads. Biofortified wheat varieties may enable higher fibre white breads [54,55], but the primary approach is to incorporate wholegrains, seeds and fortification with processed fibres [56]. Most commercial bread brands now offer a combined white-wholemeal loaf, referred to here as ‘combi’ bread. Some brands are developing higher fibre breads designed to appeal to people accustomed to white bread [56]. However, despite increasing appreciation of more fibre-rich breads in the UK [49], white bread remains a default staple for many Britons and retains a somewhat totemic status in the British diet [47,57], especially among lower income consumers [58]. Accordingly, large-scale consumption of nutritionally poorer white bread implies an opportunity cost in fibre consumption [47,49,59]. According to our rough calculations based on National Diet and Nutrition Survey and bread sales data, shifting national white bread consumption to combi bread could close the fibre gap by about 13% (approx. 0.75 g per day) in primary school aged children and 10% in secondary aged children (approx. 0.9 g per day). Switching to high-fibre (minimum 6 g 100 g^−1^) bread would have a larger effect, around 33% (1.9 g) in primary and 25% (2.3 g) in secondary aged children.

H3 is working closely with Leeds City Council to map the existing provision of breakfast in schools across Leeds. Despite government buying standards dictating that 50% of bread served in schools must contain a minimum of 3 g fibre 100 g^−1^ and 25% must contain 6 g 100 g^−1^ [60], enforcement in schools is uncertain [61]. Returns from our recent (2024) survey of 175 schools across Leeds indicate that over 97% of school breakfasts include bread or bagels, with 79% of schools offering refined, white versions at breakfast, and 39 schools (26%) not offering any wholegrain or ‘50 : 50’ breads alongside. Around three-quarters of school breakfasts were not fully compliant with school food standards. The full Leeds school breakfast survey report can be viewed here: https://www.schoolwellbeing.co.uk/uploads/optimadmin/document/document/562/School_Breakfasts_in_Leeds_Report__2_.pdf.

**Figure 1.**
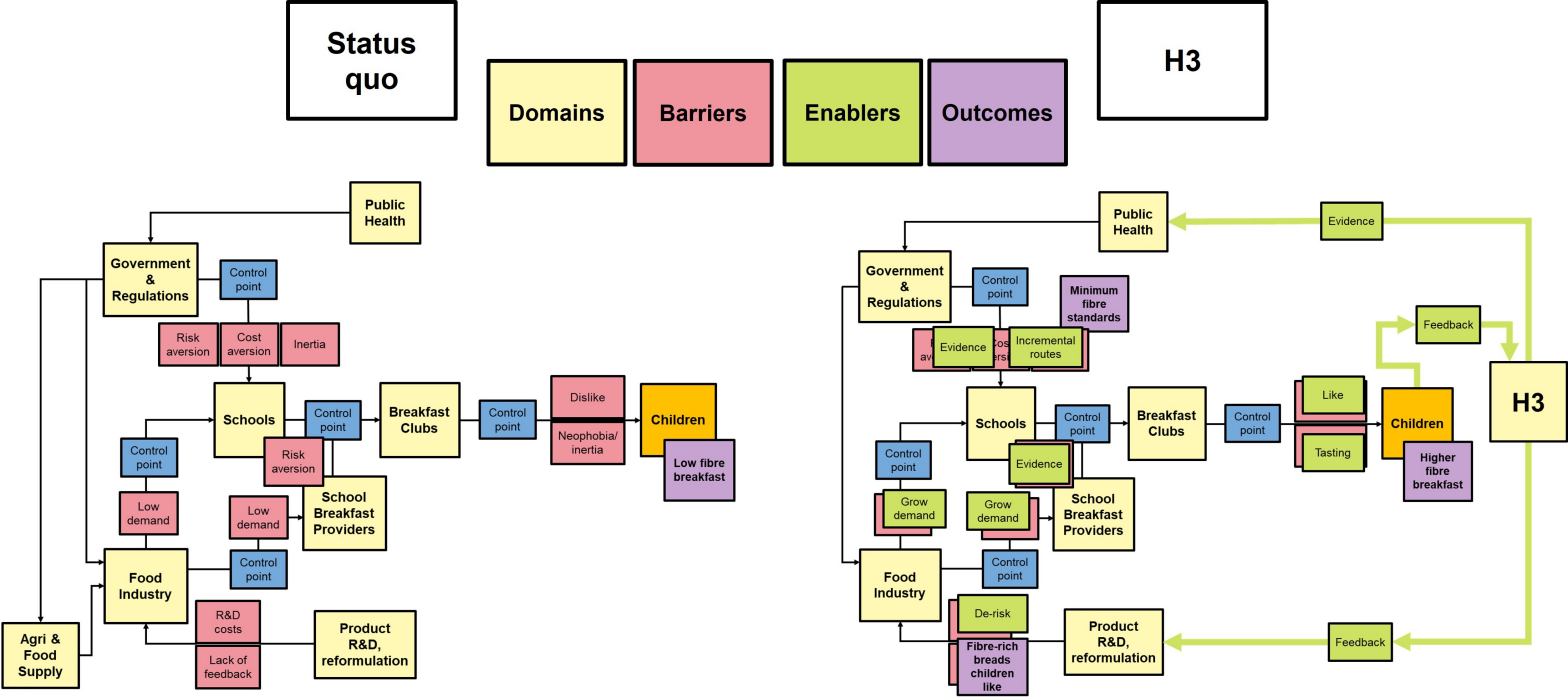
Theory of change. The status quo (left) is large-scale provision of low-fibre white bread in schools, sustained by a number of actual or perceived barriers to change at various control points in the school food system. Our H3 research (right) examines these barriers and aims to supplant or overcome them with enablers, so as to unblock decision-making and product design concurrently across the control points and active decision makers/gatekeepers.

Many UK children prefer ‘smooth’ white bread to wholemeal and seeded breads, probably owing to a combination of familiarity and sensory appeal [62]. Therefore, given the option of white bread, most children will choose it [62]. Purchasing of other breads may then look like a waste, incentivizing financially pressured schools to skirt provision requirements [43]. For all these reasons, replacing white bread in schools is perceived as a difficult and high-risk endeavour, and this perception presents a significant barrier to change across the food system (see figure 1). It is difficult to find published evidence to this effect (as is often the case with default decisions), but we have encountered this view frequently in schools, and other reasons for the current policy are not apparent.

Schools and providers are reluctant to make changes perceived as likely to increase waste and compromise their basic purpose of feeding all the children on budget. Manufacturers may be disincentivized to develop higher fibre breads aimed at children owing to unreliable demand (see box 1). Policymakers may be dissuaded by perceived difficulty and political controversy. The result is a perverse status quo wherein UK schools are feeding children large quantities of lower fibre refined white bread, even as public health research identifies low fibre consumption as a significant contributor to dietary ill health [13,63]. Children are likely to grow up accustomed to white bread as the norm, and to carry this habit into adulthood and the next generation [64]. Thus, children and their preferences are assumed to be the key barrier to change, even though it is adult gatekeepers who determine their food options. Here, we question this framing, conducting studies to examine whether children’s preferences really constitute such a major barrier. Conversely, we take the view that the key problem is that the school food system provides children with lots of low-fibre white bread, which the children then eat.

Our proposed solution is at the system level, i.e. for schools to stop providing low-fibre white bread and instead provide *only* more nutritious breads that children *like enough to eat*, even if they like white bread more. Some 20% of Leeds schools (primary and secondary) have implemented this approach in their breakfasts (according to our survey), and Magic Breakfast now supplies only bread that is a ‘source of fibre’ (>3 g 100 g^−1^). There is probably an overlap here, i.e. those schools being in part Magic Breakfast provided schools, but we do not currently have data to substantiate this. However, there is limited evidence to support the success of this approach from the perspective of children. Hence, we conducted H3 work in primary schools to examine children’s preferences and acceptance regarding higher fibre foods.

Our theory of change is illustrated in figure 1. The combination of the default status of white bread and the impression that ‘the children will only eat white bread’ means that school children are provided with white bread as standard. Given their existing familiarity and preference, most children then eat mostly white bread at school, and rarely choose higher fibre breads when white is available. Policymakers are then reluctant to disturb a ‘working’ status quo, while public health advocates bemoan the low fibre content of children’s diets and research how to change children’s choices and preferences. Industry is not incentivized to produce higher fibre breads suited to children’s tastes. Thus, at the system level, these barriers reinforce one another. Our research has found little knowledge of or interest in fibre consumption and its health benefits in children, so the impetus for change will likely need to be applied externally (see Box 4). Our suggestion is to focus on choices of policymakers and school food gatekeepers (e.g. headteachers, school food providers), rather than children, with a simple message: we should not provide children with foods we do not want them to eat, and we should instead provide only foods we do want them to eat. The key data point here is that the children *will not* only eat white bread (they just prefer white bread); we were expecting much more resistance from children, but did not encounter it.

Box 4. **Children’s perspectives.**

We conducted focus groups and tasting activities with over 150 young people in secondary schools to better understand their knowledge of fibre and its role in dietary health (publication in preparation). In accordance with national statistics, home diets are often quite low in fibre, and school food standards do not mandate a fibre-rich diet at school. Therefore:

— many young people are not accustomed to eating high-fibre foods.

Most young people had heard of dietary fibre but knew little about it. Few were aware of its health benefits, and knowledge of which foods are sources of fibre was limited. Therefore:

— many young people are not interested or empowered to seek out high-fibre foods;
— ascertaining accurate estimates of fibre consumption from surveys is unlikely;
— basic nutrition education about the importance of fibre is lacking; and
— interventions to quickly increase fibre consumption should not rely on children’s choices.

## 3. Higher fibre breads in primary school breakfast clubs

Scoping work began in two primary school breakfast clubs (each usually hosting approx. 35 children per day), in collaboration with school breakfast club provider Magic Breakfast (charity number 1 102 510 in England and SC048202 in Scotland). We first pilot-tested our methods, establishing a simple procedure for measuring children’s liking and breakfast choices and assessing children’s opinions of foods in the Magic Breakfast range. Concurrently, Magic Breakfast began supplying their breakfast clubs with a white-wholemeal blended bread (50% white, 50% wholemeal). This bread was widely accepted by the children, and when toasted is visually similar to white toast. Inspired by the children’s openness to this combined half white-half wholemeal bread (‘combi bread’), we next introduced the Prograins range of higher fibre breads developed by ABMauri, plus some higher fibre breakfast cereals and fresh fruit [65]. Research was conducted in two different primary school breakfast clubs in Leeds, each hosting approximately 30–40 children per day. For further details, see our published report of this work [65].

The ‘Protein Powered’ bread was fortified with pulse flours, providing both fibre (5.8 g 100 g^−1^) and protein. ‘Heritage Spelt’ was a wholemeal-white spelt flour combi bread (fibre 4.1 g 100 g^−1^). ‘Sprouted Multiseed’ (fibre 6.7 g 100 g^−1^) had a darker crumb and contained sprouted seeds. ‘BARLEYmax® High Fibre’ (fibre 11.3 g 100 g^−1^) had a light crumb and wholegrain flakes. The former two breads were smooth, while the latter two were ‘bitty’ (the word most often used by children to describe bread with particulate seeds and wholegrains). Different approaches to fibre enrichment may have different digestive effects [66], and other aspects of a food may also influence glucose release, e.g. protein content [67], which might affect satiety and energy release across the morning with implications for concentration and performance. Therefore, we considered it useful to examine the digestive properties of these novel breads.

We examined—via *in vitro* digestive analysis—the glucose release curves of Prograins breads versus ‘standard’ white, 50% white/wholemeal ‘combi’ bread and wholegrain comparators, all supplied by ABMauri [65]. The Prograins breads yielded flatter glucose release curves with lower peaks and longer glucose release (relative to the ‘standard’ breads). Glucose is essential to cellular activity and human performance in physical and mental activity [68], so extended release is desirable to fuel children’s participation in school activities for the approximate 4 h from breakfast until lunch. By contrast, the ‘standard’ white bread yielded a fast, high glucose release peak that dropped rapidly, providing little glucose after 2 h, while the standard combi and wholemeal breads provided a somewhat lower initial spike and longer glucose release. Higher fibre was associated with longer, steadier glucose release, but other factors such as particulate and protein content interacted such that this relationship was not linear; the highest fibre bread (‘BARLEYmax® High Fibre’) did not have the flattest curve, nor the lowest peak, nor the lowest predicted glycaemic index, but was nonetheless preferable to all the ‘standard’ control breads [65].

Equally, different approaches to fibre enrichment are likely to differentially affect sensory properties and hence liking [56]. Previous research has found that ‘smooth’ breads are more widely liked than ‘bitty’ breads by children, and that lighter crumb is preferred to darker [69]. To examine children’s liking and acceptance, we introduced the Prograins breads to two primary school breakfast clubs and measured children’s liking of these breads using a simple ‘Taste&Rate’ activity. Recent focus group research has found that children reported being open to tasting activities [70], and recent experimental results have affirmed this [71]. Children tasted the breads and rated them ‘yum, ‘yuck’ or ‘OK’. The inclusion of ‘yuck’ has been questioned as pejorative, but it is important that dislike is actively validated to encourage children to express their actual opinions rather than just responding to expectations.

This activity was intended both to familiarize children and enable them to identify their preferred breads before being put in the position of choosing them for breakfast, and to inform us about the pattern of liking for the different breads. Children’s preferences were varied and individual. The most challenging (dark and bitty) Sprouted Multiseed bread was least frequently liked, but still rated yum by 29% of children and yum-or-OK by 64%. As expected, the ‘smooth’ breads were well liked. The light coloured but somewhat ‘bitty’ BARLEYmax® High Fibre bread was also well liked. These other three breads were liked similarly (collectively 67% yum, 94% yum or OK) to the combi bread (70% yum, 95% yum or OK).

Next, we observed the extent to which children chose the Prograins breads when offered alongside the usual breakfast club provision. At baseline, with only combi bread on offer (in addition to cereals), on average, half a slice per child was chosen. When the Prograins breads were available, this dropped to under a third of a slice, while children chose on average three-quarters of a slice (per child) of the intervention breads. Of the latter, the children chose 32% Heritage Spelt, 30% Protein Powered, 20% BARLEYmax^®^ High Fibre and 17% Sprouted MultiSeed. Notably, the visibly ‘bitty’ breads (Sprouted Multiseed and BARLEYmax® High Fibre) were chosen least frequently. We concluded that if a single bread is offered, then a bread with a light visual aspect and smooth texture is likely to be most widely accepted. However, if multiple breads are provided, then darker and more seedy/bitty breads may appeal to a worthwhile minority of children.

## 4. Natural experiment: replacing low-fibre white bread in school breakfast provision

We conducted a naturalistic school-based experiment providing daily breakfast to a primary school in East Liverpool between February and July 2024. At the starting point of the intervention, the school had been providing a simple universal breakfast of toasted white bread with non-dairy spread since September 2023. The aim was to assess the feasibility of *replacing* white bread provision with higher fibre breads. We offered to sponsor bread provision, on the proviso that white bread would be removed from the provision and that all the bread choices offered would be good sources of fibre. Here, we report initial results of our evaluation of this intervention; more detailed reporting is forthcoming in a dedicated publication looking into potential effects on pupil well-being and attendance.

The ABMauri Prograins range was not at the stage of mass production and could not be supplied at the volume needed for a six month intervention. Jackson’s of Yorkshire kindly agreed to provide four fibre-enriched breads from their commercially available ‘Champion Bloomers’ range. ‘Bloomin’ Both’ bread is a 50% combination of white and wholemeal flour, fortified with powdered linseeds (fibre 5.7 g 100 g^−1^). ‘Multigrain Brown’ is a malted brown loaf enriched with wholegrains (fibre 4.4 g 100 g^−1^), ‘Super-seeded’ is a brown loaf enriched with seeds (fibre 7 g 100 g^−1^), and ‘Multiseed’ is a white loaf enriched with seeds (fibre 6 g 100 g^−1^). We refer to these as ‘Jackson’s breads’ throughout. These breads are cut into 45 g slices. Teachers expressed some doubt about whether children would accept these higher fibre breads.

**Figure 2.**
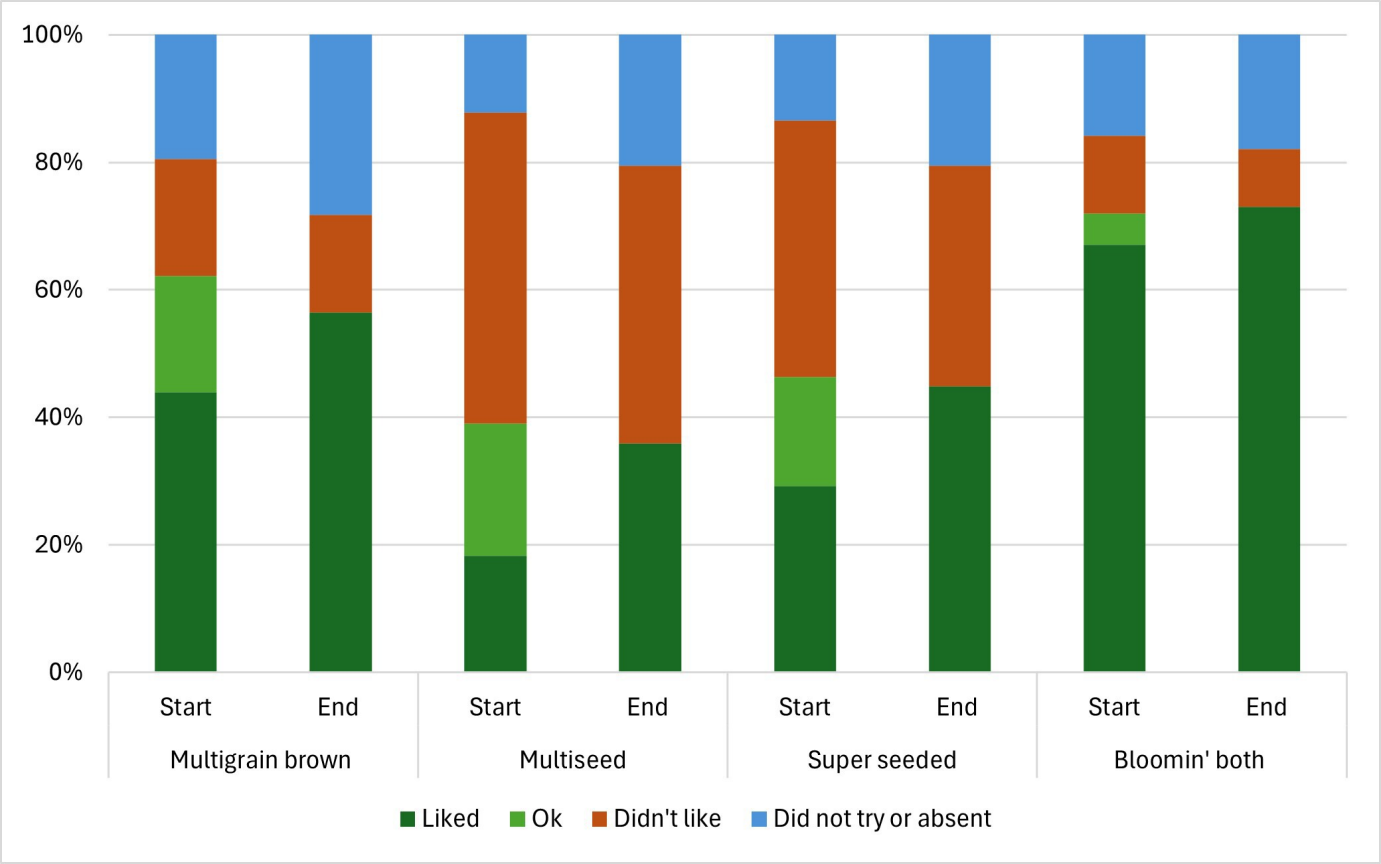
Taste&Rate ratings for high-fibre Jackson’s bread options before (*n* = 82) and after (*n* = 78) six months of school breakfast provision exposure.

At the beginning of the intervention period (February 2024), the new Jackson’s breads were first introduced to the children through a Taste&Rate activity in class, where each child could taste a one-quarter slice of four breads. As expected, the Bloomin’ Both combi bread was the most widely liked, while the darker and/or seeded breads were liked by sizable minorities of children. We repeated the Taste&Rate activity near the end of the intervention (July 2024) after six months of exposure to Jackson’s breads. Complete before and after data for school years 1, 4 and 5 were obtained. These data indicate increased liking for all the breads post-intervention. The data appear to suggest that children initially rated a bread ‘OK’ but had reached firmer conclusions, with most of them switching to liking. However, individuals were not tracked between baseline and follow-up, so we cannot demonstrate individual-level changes. Aggregate class data are shown in figure 2.

We also collected insights from teachers regarding the breakfast provision. In contrast to teacher expectations, children usually ate all the toast available, and leftovers were rare; ‘the children were excited to get their toast in the morning*’,* and *‘*all the children found at least one bread they liked’. The smooth textured Bloomin’ Both bread was most popular by some margin, but a worthwhile minority of children also liked and ate the wholegrain and seeded loaves. Typical fibre content of a slice of white bread is approximately 1 g, whilst the most popular Jackson’s bread (Bloomin’ Both) contains 2.6 g slice^−1^. Thus, this simple change in the breakfast breads offered to children increased breakfast fibre by approximately 1.6 g slice^−1^ consumed, which is approximately 25% of the average 6 g ‘fibre gap’ in 4−10 years old children [72].

Initial results from this natural experiment suggest that children’s preferences and dietary inertia are not the insurmountable barrier they are often assumed to be. Replacing white bread with a choice of higher fibre alternatives was feasible, even using rather ‘grown-up’ bread products, in a hard-pressed school serving a community with high levels of disadvantage. Schools are already obliged to meet certain nutritional standards for bread provision, but these are not very stringent nor strongly enforced. Strengthening and enforcing requirements such that all bread served in schools must be a ‘source of fibre’ (and/or minimum wholegrain requirements) would not be a radical step given the public health need. While UK adults may be settled in their white bread eating habits [54], we found that children were quite open to higher fibre breads.

Strategic use of school food offers a policy lever by which generational shifts in eating habits and food culture can be approached, and school food procurement offers a valuable policy lever to influence food system incentives [10]. Larger scale pilots and assessment should be the next step, ideally as part of the national school breakfast pilot programme. These should address some open questions highlighted by our studies.

First, we think that the Taste&Rate activity may have helped to smooth the transition from breakfast provision of white to higher fibre breads. However, to date, we have not conducted a controlled experiment comparing breakfast service intervention with versus without an introductory Taste&Rate activity. Controlled studies should be conducted to understand if the Taste&Rate activity is important or dispensable to outcomes. The Taste&Rate ratings should be treated with caution and as approximate. The primary purpose of the activity was to introduce the children to the new breads in a conducive context [71] and give *them* a chance to see which ones they liked. Second, the resources available for this study were limited, and the school environment restricted what data could be collected. More intensive data collection on daily consumption, as well as better pupil coverage, would be valuable. Third, larger samples representative of the national demographic are needed. Fourth, while the Jackson’s breads were successful, other breads more tailored to children’s tastes might be even more successful and have a more reliably broad appeal. Our innovation work with ABMauri to develop bread products with high fibre content, broad sensory appeal to children and good suitability to the school breakfast context is a step in this direction.

## 5. The H3 bagel for school breakfasts

It became apparent during repeated attendance at school breakfast provision settings that white bagels (New York Bagel Company, fortified with wheat fibre to give fibre 3.6 g 100 g^−1^) were particularly popular in school breakfast clubs, when available. Staff and children explained that bagels hold their texture better than toast, which becomes soggy. This is important because toast/bagels are often prepared in a large batch at the beginning of the breakfast club, and so may sit for some time before consumption, becoming cold. We also found that the BARLEYmax® HighFibre bread was well liked by children who tried it, but some children were put off from choosing it by the visible ‘bits’ of grain flakes. The biofortified BARLEYmax® grain was conventionally bred for high fibre content [73], and has been shown to have desirable glycaemic index (GI) [74] and gut microbiome effects [75]. We reasoned that a bagel made with a more refined BARLEYmax® product (i.e. less ‘bitty’) could be a suitable product for a high fibre, high acceptance bread for school breakfast clubs.

**Figure 3.**
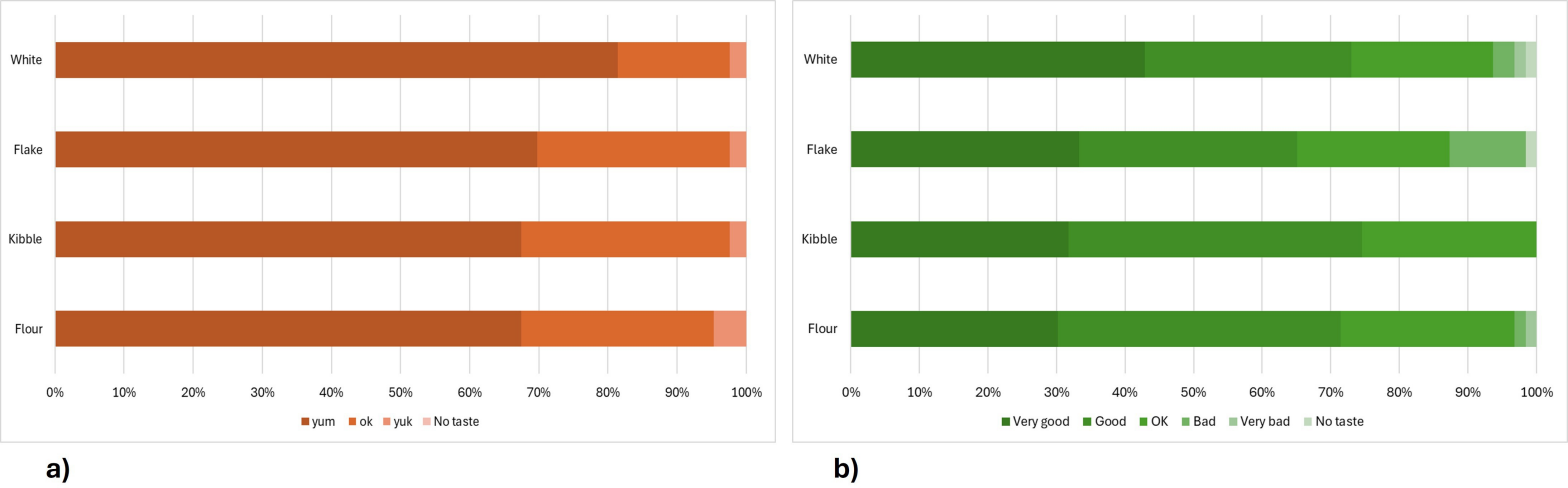
Taste&Rate ratings for the H3 bagel prototypes among (a) primary school children (*n* = 43) and (b) secondary school children (*n* = 63).

In collaboration with ABMauri, we developed three prototype bagels with three different levels of texture/bitty-ness: a smooth bagel made with only flour, a semi-smooth bagel made with kibble and a more ‘bitty’ bagel made with flaked grain. For a baseline comparator, we used a white bagel fortified with wheat fibre. All of these contained 6 g fibre 100 g^−1^. To assess children’s liking of these prototypes, we conducted Taste&Rate sessions in school breakfast clubs in primary (*n* = 43 children) and secondary (*n* = 63 children) schools. The kibble recipe was the most popular of the BARLEYmax® prototypes and the best option for the baking process, as the fine flour increases crumb density, while the flakes disturb gluten strand formation. In primary school children, 67% rated it yum, and 97% yum or OK (see figure 3). In secondary school, 75% rated it very good or good, with the other 25% rating it OK. The other prototypes were slightly less well liked. These initial results show that it is possible to develop high-fibre bread products which children like, even with low salt and no added sugar. The white bagel was also well liked, offering a lower cost alternative if its digestive properties are equally desirable. We are currently conducting *in vitro* digestion analysis of the glucose release profiles for these prototype bagels.

With over nine million children attending school in England [76], the school breakfast market is potentially very large, especially considering current UK government proposals for free-of-charge universal primary school breakfast provision [1]. Large-scale school breakfast provision presents an important opportunity to increase fibre consumption in children and adolescents. Incrementally increasing fibre requirements in the school food standards for bread would provide the market to drive industry innovation in high-fibre bread products designed for children, and concurrently increase children’s fibre consumption. If high-fibre wholegrain bread products like the ‘H3’ bagel can be established as standard staples in universal school breakfast provision, a significant reduction in the nationwide ‘fibre gap’ in children would result.

## 6. Conclusion

If schools are to meet their full potential as vectors of food system transformation, cultural value and an investment mindset will need to be assigned to school food provision and education. In the meantime, policy choices to raise the nutritional baselines for school food provision offer ‘low hanging fruit’, which could rapidly and widely improve children’s dietary intake (as exemplified here with fibre consumption). Price point is an issue with innovative high-fibre breads, but for a first step industry standard breads made with half white and half wholemeal flour are often price matched to white bread within the brand. Magic Breakfast has replaced white bread with such combined breads, encountering little resistance from children; this could be replicated across the entire school food system at almost no cost. Higher fibre and/or wholegrain minimum requirements for bread in schools could incentivize the food industry to develop affordable, compliant products to children’s tastes in order to access the school food market. Overall, this reflects the school food choice architecture strategy, ‘only offer healthy options’. The responsibility for providing nutritious and delicious school food is placed on the adult gatekeepers (from policymakers to local authorities, headteachers and school food providers), rather than upon children choosing the 'good' option. It is then essential for public health advocates to convince—with evidence—these adult gatekeepers operating in school food provision and those with regulatory oversight that improvements in minimum nutrition requirements for schools are feasible, worthwhile and acceptable to children. The current work provides initial evidence for the case of school breakfasts with higher fibre breads and demonstrates the feasibility of child-centred development of high-fibre bread products that children enjoy. Larger scale trials should be the next step to gather stronger evidence from larger samples representative of the national school population, identify potential issues such as parent pushback and increased food waste, and establish whether our findings here generalize.

## Data Availability

Data referenced is available as the electronic supplementary material [77]. Data for the Leeds Breakfast Survey is owned by Leeds City Council.
